# Surgical approach to solid pseudopapillary neoplasms of the proximal pancreas: minimally invasive vs. open

**DOI:** 10.1186/s12957-019-1684-7

**Published:** 2019-09-12

**Authors:** Emmanuel II Uy Hao, Seoung Yoon Rho, Ho Kyoung Hwang, Jae Uk Chung, Woo Jung Lee, Dong Sup Yoon, Chang Moo Kang

**Affiliations:** 1 Department of Surgery, University of the Philippines-Philippine General Hospital, Taft Avenue, Manila, Philippines; 2 Division of Hepatobiliary and Pancreatic Surgery, Department of Surgery, Yonsei University College of Medicine, Seoul, South Korea; 3 Pancreatobiliary Cancer Center, Yonsei Cancer Center, Severance Hospital, Seoul, South Korea; 4 Department of Surgery, National Health Insurance Service Ilsan Hospital, Goyang, South Korea; 5 Pancreatobiliary Cancer Clinic, Department of Surgery, Gangnam Severance Hospital, Yonsei University College of Medicine, Seoul, Korea

## Abstract

**Background:**

Solid pseudopapillary neoplasms (SPN) of the pancreas are rare pancreatic neoplasms where complete resection is the cornerstone in management. It has been demonstrated in previous studies that minimally invasive surgical approaches are effective management options in treating SPNs of the distal pancreas. The purpose of this study is to evaluate the feasibility of minimally invasive surgery in treating SPNs of the uncinate, head, and neck of the pancreas.

**Methods:**

Data from 2005 to 2017 at Severance Hospital of the Yonsei University Health systems in Seoul, South Korea, were retrospectively collected for 25 patients who were diagnosed with SPN of the uncinate, head, and neck of the pancreas and who underwent curative resection. Three groups of patients were considered, depending on the year of surgery, in order to determine trends in the surgical management of SPN. The patients were also divided into two groups corresponding to the type of operation done (minimally invasive surgery vs. open surgery). Perioperative patient data, including age, gender, body mass index (BMI), tumor size, and operation done, were compared and analyzed statistically. Long-term nutritional effects were measured using the Controlling Nutritional Status (CONUT) scoring system.

**Results:**

There were no statistically significant differences in age, gender, BMI, symptomatic presentation, operation type, tumor size, and tumor stage between the three time periods. In comparing between minimally invasive and open surgery, there were no statistically significant differences in age, gender, symptomatic presentation, BMI, tumor size, preoperative stage, type of operation, operation time, pancreatic duct size, post-operative pancreatic fistula (POPF) grade, death associated with disease, recurrence, pathological parameters, and change in CONUT score. There was a significant difference in tumor size (4.5 ± 1.8 vs. 2.6 ± 1.0 cm, *p* = 0.004), blood loss (664.2 ± 512.4 vs. 277.7 ± 250.8 mL, *p* = 0.024), need to transfuse (33% vs. 0%, *p* = 0.023), hospital length of stay (27.4 ± 15.3 vs. 11.5 ± 5.3 days, *p* = 0.002), and complication rate (75% vs. 30.8%, *p* = 0.027) between the two groups.

**Conclusions:**

In appropriately selected patients with SPNs of the uncinate, head, and neck of the pancreas, a minimally invasive surgical approach offers at least equal oncologic and nutritional outcomes, while demonstrating decreased complications and decreased hospital length of stay compared with that of an open surgical approach.

## Background

Neoplasms of the pancreas are the fourth most common cause of cancer-related death globally [1]. These tumors are often difficult to detect due to their location; most patients present with non-specific symptoms or clinicians discover them on incidental radiographs for different conditions [2, 3]. Solid pseudopapillary neoplasm (SPN) of the pancreas, also referred to as Frantz’s tumor, is rare, comprising 1–2% of all pancreatic tumors [4–6]. SPNs of the pancreas are usually diagnosed in females who are in their third to fourth decade of life and this tumor carries a good prognosis, with a 5-year survival rate of up to 97% [6–9]. SPN is considered to be a tumor with low malignant potential; however, up to 10–15% of cases have been reported to be aggressive and can metastasize to the liver and/or peritoneum [10]. Despite its malignant potential, resections with no microscopically detected cancer cell at the resection margin (R0) and en bloc resections have been reported to improve overall survival and disease-free survival [11, 12].

The distal pancreas is the most common site of occurrence, but with advancements in imaging techniques and practices, SPNs located at the uncinate, head, and neck of the pancreas, referred collectively henceforth as the proximal pancreas, are now being increasingly reported [9, 13]. A previous study compared the outcomes of minimally invasive (MI) and open distal pancreatectomies in patients with SPN where the outcome favored minimally invasive surgical approaches (laparoscopic and robot-assisted) [14]. As such, the need to investigate surgical approaches for SPNs of the proximal pancreas is needed in order to provide the surgeon with viable options in the management of all SPNs of the pancreas.

Therefore, the purpose of this study was to compare the perioperative short-term and long-term outcomes of minimally invasive (laparoscopic and robotic) and open surgical approaches of the proximal pancreas in patients with pathologically confirmed SPNs.

## Methods

### Data collection

Patients’ medical records were retrospectively reviewed to identify those who underwent either open or MI pylorus-preserving pancreaticoduodenectomy (PPPD), classic Whipple procedure (PD), or central pancreatectomy (CP) for pathologically confirmed SPNs between 2006 and 2017 at the Severance Hospital of the Yonsei University Health System in Seoul, South Korea. Data representing the clinicopathological characteristics, such as age, gender, tumor size, and location, were retrospectively collected. Patients who underwent extensive surgeries or combined resections were excluded from this study in order to avoid selection bias in the comparative analysis between MI and open surgical approaches.

For the assessment of long-term nutrition, the Controlling Nutritional Status (CONUT) scoring system was used. The CONUT score utilizes serum albumin, cholesterol, and total lymphocyte count as parameters to measure the nutritional status of patients and has been shown to be predictive of survival in colorectal neoplasms [15, 16] and hepatocellular neoplasms [17]. We stratified the study population into three groups according to the year when the surgery was performed (e.g., 2006–2009, 2010–2013, 2014–2017) to determine the chronological trends of clinical characteristics in patients with SPNs. This study protocol was approved by the local institutional review board, with the IRB number 4-2018-0001.

### Statistical analysis

IBM SPSS Statistics, version 23 (SPSS Inc., Chicago, IL, USA) was used for the statistical analyses. Continuous variables were presented as mean ± standard deviation and categorical variables were represented as percentage or frequency. Student’s *t* test was performed to compare the continuous variables, and Fisher’s exact test or the chi-squared test was used to compare categorical data between approaches.

## Results

### Chronological changes of clinical characteristics according to period

From 2006 to 2017, 98 patients underwent surgery for SPN at the Severance Hospital of the Yonsei University Health Systems. Of the 98 patients, 25 were diagnosed with SPN at the proximal pancreas and were included in this study. There were no statistical differences in the age, gender, BMI, and symptoms across the three periods (Table 1). There was an increasing trend of patients undergoing minimally invasive procedures from the first time period up to the third time period; however, this was not statistically significant (*p* = 0.269). Moreover, the neoplasm stage at presentation was not statistically significant; however, it was noted that the size of tumors was smaller during the period from 2014 to 2017 compared to the period from 2010 to 2013.

**Table 1:** Summary of data per time period

	2006–2009	2010–2013	2014–2017	*p* value
Number	7	11	7	
Age (years)	39.43 ± 12.78	35.91 ± 15.42	40.71 ± 15.55	0.775
Gender (F:M)	7:0	9:2	6:1	0.500
BMI (kg/m^2^)	21.71 ± 2.87	22.82 ± 2.36	22.43 ± 2.88	0.694
Symptomatic (yes/no)	2/5	5/6	2/5	0.997
Operation type (MI/open)	2/5	5/6	5/2	0.269
Operation (PPPD/CP)	3/4	8/3	5/2	0.389
Tumor size (cm)	3.63 ± 1.91	4.12 ± 1.82	2.44 ± 0.78*	0.126
Stage				0.833
Ia	2 (28%)	2	2 (28%)	
Ib	5 (72%)	9	5 (72%)	

Data are presented as number, mean ± standard deviation, or number (percentage)

*F* female, *M* male, *BMI* body mass index, *MI* minimally invasive, *PPPD* pylorus preserving pancreaticoduodenectomy, *CP* central pancreatectomy

*There was a statistically significant (*p* = 0.017) decrease in tumor size noted between periods 2010–2013 and 2014–2017

### General patient characteristics

Of the 25 patients included in this study, 22 were female and three were male (female to male ratio was 7.3:1). On average, the patients were 38.2 ± 14.3 years old and had a BMI of 22.4 ± 2.58 kg/m^2^. Additionally, the mean tumor size was 3.51 ± 1.72 cm with a mean pancreatic duct size of 1.29 ± 1.00 mm. Nine of the patients presented with symptoms associated with the tumor, while the tumor was an incidental finding in the remaining 16 patients. Six (24%) patients presented as stage Ia and 19 (76%) as stage Ib. Twelve patients underwent an open surgical procedure and 13 underwent a minimally invasive surgical procedure, two of which underwent robotic surgery. Nine patients underwent CP and 16 underwent PPPD. There were no reported cases of patients who underwent PD. The patients were followed up for an average of 48.0 ± 31.9 months.

### Comparative analysis between MI and open surgical approaches for SPN of the proximal pancreas

There were no statistically significant differences between patients who underwent open versus patients who underwent MI surgeries in terms of age, gender, symptomatic presentation, BMI, preoperative stage, type of operation, operation time, pancreatic duct size, and post-operative pancreatic fistula (POPF) grade (*p* > 0.05 for all; Table 2). All patients underwent an R0 resection and all remnant pancreata were described to be soft on palpation. In contrast, tumor size, blood loss, need for transfusion, hospital length of stay, and complication incidence were significantly greater in open surgery compared to MI surgery (*p* ≤ 0.027 for all).

**Table 2:** Perioperative comparison between minimally invasive and open surgeries

	Minimally invasive (*n* = 13)	Open (*n* = 12)	*p* value
Age (years)	41.0 ± 13.5	35.3 ± 15.4	0.326
Gender (F:M)	11:2	11:1	0.588
Symptomatic (yes/no)	3/10	6/6	0.161
BMI (kg/m^2^)	22.8 ± 2.6	21.9 ± 2.5	0.380
Tumor size (cm)	2.6 ± 1.0	4.5 ± 1.8	0.004*
Preoperative stage			0.645
Ia	4 (30.8%)	2 (16.7%)	
Ib	9 (69.2%)	10 (83.3%)	
Operation			0.411
PPPD	7 (53.8%)	9 (75%)	
CP	6 (46.2%)	3 (25%)	
OR time (min)	417.2 ± 115.6	413.9 ± 132.4	0.949
Blood loss (mL)	277.7 ± 250.8	664.2 ± 512.4	0.024*
Need for transfusion (yes/no)	0/13	4/8	0.023*
Pancreas description (soft/hard)	13/0	12/0	
Pancreatic duct size (mm)	1.15 ± 0.83	1.43 ± 1.2	0.496
Resection type	All R0	All R0	
Hospital length of stay (days)	11.5 ± 5.3	27.4 ± 15.3	0.002*
Complication incidence	4 (30.8%)	9 (75%)	0.027*
POPF (grade A/grade B)	2 (15.4%)/2 (15.4%)	3 (25%)/1 (8.33%)	0.465

Data are presented as number, mean ± standard deviation, or number (percentage)

*F* female, *M* male, *BMI* body mass index, *PPPD* pylorus preserving pancreaticoduodenectomy, *CP* central pancreatectomy, *OR* operating room, *R0* no microscopically detected cancer cells at the resection margin, *POPF* post-operative pancreatic fistula.

*Statistically significant difference between surgical approaches at *p* < 0.05

### Histopathologic outcomes

We compared the histopathologic outcomes of the patients based on their surgical approach. The collected data is presented in Table 3. During the follow-up period (median, 57 months; range, 4–120 months), none of the patients in this study experienced tumor recurrence or death as a result of the disease. All pathologic resection margins were negative for disease. Further, a review of the microscopic pathologic parameters (capsular invasion, lymphovascular invasion, perineural invasion), as well as the post-operative stage, revealed that there were no significant differences between the surgical approaches.

**Table 3:** Histopathologic outcomes of minimally invasive and open surgeries

	Minimally invasive (*n* = 13)	Open (*n* = 12)	*p* value
Death associated with disease	None	None	
Recurrence	None	None	
Resection margin	All negative	All negative	
Capsular invasion	3 (23.1%)	2 (16.7%)	0.698
Lymphovascular invasion	1 (7.69%)	0	0.327
Perineural invasion	3 (23.1%)	2 (16.7%)	0.689
Stage			0.409
Ia	4 (30.8%)	1 (8.33%)	
Ib	6 (46.2%)	9 (75%)	
IIa	3 (23.1%)	2 (16.7%)	

Data are presented as number (percentage)

### Long-term nutritional assessment

The CONUT scoring system was used to determine the nutritional status of patients prior to surgery and 6 and 12 months after surgery. Nearly all of the patients had improvements, and none experienced worsening, in nutritional status (Table 4). No significant changes in nutritional status were detected between the surgical approaches at 6 and 12 months after surgery (*p* > 0.05 for all, Table 4).

**Table 4:** Changes in CONUT score of patients undergoing surgery of the proximal pancreas

CONUT score for severity of malnutrition	Pre-operative *n* (%)		6 months post-operative *n* (%)		12 months post-operative *n* (%)	
	Minimally invasive (*n* = 13)	Open (*n* = 12)	Minimally invasive (*n* = 13)	Open (*n* = 12)	Minimally invasive (*n* = 13)	Open (*n* = 12)
Normal (without deficit)	0	0	9 (69%)	9 (75%)	11 (85%)	10 (83%)
Mild	7 (54%)	4 (33%)	4 (31%)	3 (25%)	2 (15%)	2 (17%)
Moderate	5 (38%)	6 (50%)	0	0	0	0
Severe	1 (8%)	2 (17%)	0	0	0	0

*CONUT* Controlling Nutritional Status

## Discussion

In a previous study, we have reported the surgical outcomes of minimally invasive distal pancreatectomy for SPN of the pancreas [14]. There are also several literatures reported [18–22] showing favorable perioperative outcomes in managing SPN of the distal pancreas; however, minimally invasive surgical procedures (PPPD or CP) for SPN in the proximal pancreas seems rare.

Patients who underwent open or MI surgeries for SPN at the proximal pancreas showed no statistically significant differences in terms of gender, age, BMI, pancreas characteristics, operation time, and American Joint Committee on Cancer (AJCC) cancer stage. However, it was demonstrated that patients with MI surgery had a smaller tumor size at presentation (mean, 2.6 vs. 4.5 cm, respectively), which may have been a factor in deciding which surgical approach to perform in managing SPN of the proximal pancreas.

The study results showed statistically significant differences in length of hospital stay, complication incidence, blood loss, and transfusion requirement, all of which favored the MI surgical approach. These parameters are often used when comparing surgical procedures and their possible adverse effects on patients. Needless to say, shorter hospital stay, decreased blood loss, and lower need for transfusion are all positive aspects that advocate for the recommendation of MI surgery in selected patients with SPN of the proximal pancreas. These findings coincide with those of Torres et al. who reported a successful laparoscopic pancreaticoduodenectomy in a 19/F with SPN who was discharged after 6 days without complication [23]. Similarly, Senthilnathan et al. reported five successful laparoscopic pancreaticoduodenectomy operations in their 8-year experience [24].

It should also be noted that there was no residual tumor after the operation and that all pathological reports indicated margin-free tumors in all cases. This entails that both open and MI surgical approaches can deliver comparable long-term results that are within the oncologic parameters.

The role of nutrition in improving perioperative outcomes is being recognized increasingly. Several researches describe decreased complications, as well as improved surgical outcomes, as a direct effect of improving the perioperative nutritional status of the patient [25–28]. Our study noted that there was an improvement in the CONUT score of nearly all of the patients at 6 and 12 months after surgery. However, there was no statistical difference in the change of nutritional status between the surgical approaches (*p* < 0.05). This implies that both open and MI surgical approaches can achieve quality of life goals in managing patients with SPN.

## Conclusion

The current first-line treatment for SPN of the pancreas remains to be complete excision. Studies have shown that complete excision can increase overall survival and disease-free survival. However, as with all surgical encounters, we must balance these maximal oncologic practices with minimizing complications and potential adverse outcomes. This study suggests that in appropriately selected patients with SPNs located at the uncinate, head, or neck of the pancreas, a MI surgical approach offers at least comparable oncologic outcomes as an open approach, while demonstrating decreased complications and decreased hospital length of stay. We recommend that a larger comparison be conducted in order to strengthen this conclusion.

## Data Availability

The datasets used and/or analyzed during the current study are available from the corresponding author on reasonable request.

## Abbreviations

AJCC: American Joint Committee on Cancer
BMI: body mass index
CONUT: Controlling Nutritional Status
CP: central pancreatectomy
F: female
IRB: Institutional Review Board
M: male
MI: minimally invasive
OR: operating room
PD: Whipple’s procedure
POPF: post-operative pancreatic fistula
PPPD: pylorus-preserving pancreaticoduodenectomy
R0: surgical resection with no microscopically detected cancer cells at the resection margin
SPN: solid pseudopapillary neoplasm
